# Laparoscopic Transvesical Resection of an En Bloc Bladder Cuff and Distal Ureter during Nephroureterectomy

**DOI:** 10.1100/2012/658096

**Published:** 2012-09-25

**Authors:** Stilianos Giannakopoulos, George Toufas, Charalampos Dimitriadis, Stavros Giannopoulos, Christos Kalaitzis, Athanasios Bantis, Emmanuel Patris, Stavros Touloupidis

**Affiliations:** Department of Urology, Democritus University of Thrace, Dragana, 68100 Alexandroupolis, Greece

## Abstract

*Objective*. The most appropriate technique for excising the distal ureter and bladder cuff during laparoscopic nephroureterectomy is still debated. We report our experience with a pure laparoscopic transvesical method that duplicates the long-standing open transvesical approach. *Materials and Methods*. Seven men and three women diagnosed with upper tract transitional cell carcinoma were treated with this procedure. Three intravesical ports were inserted, and pneumovesicum was established at 12 mmHg. Transvesical laparoscopic circumferential detachment of the bladder cuff and en bloc mobilization of the last centimeters of the distal ureter were performed, followed by the closure of the bladder defect. Subsequently, a nephrectomy was performed either laparoscopically or using an open flank approach. *Results*. The median age was 68.5 years. The procedure was completed uneventfully in all cases. The median operating time for distal ureter excision was 82.5 minutes (range 55–120). No complications directly related to the pneumovesicum method were recorded. The median follow-up period was 31 months (range 12–55). During the follow-up period, two patients (20%) died from the disease, and a bladder tumor developed in three cases (30%). *Conclusion*. The laparoscopic transvesical resection of the en bloc bladder cuff and distal ureter is a reliable, effective, and oncologically safe technique, at least in the midterm.

## 1. Introduction

Open radical nephroureterectomy (ONU) including en bloc excision of the distal ureter with a bladder cuff has been the standard surgical treatment for upper tract transitional cell carcinoma (TCC). Since Clayman et al. [1] reported the first case of laparoscopic nephroureterectomy (LNU) in 1991, this procedure has been used instead of ONU with increasing frequency in many centers. Studies have demonstrated that patients undergoing LNU might have decreased blood loss, less postoperative pain, a shorter duration of hospitalization, and a more rapid recovery than those patients undergoing ONU [2, 3]. Importantly, the oncologic efficacy of LNU appears comparable to that of the open surgical approach [4, 5]. The most controversial and challenging part of LNU remains the management of the distal ureter and the bladder cuff. Many different surgical approaches have been described, but no approach has been demonstrated to be superior to the others. In 2007, Cheng et al. [6] reported a transvesical laparoscopic technique using the pneumovesicum method that duplicates the long-standing open transvesical approach. Herein, we present our experience with a modification of Cheng et al.'s technique for the en bloc removal of the juxtavesical ureter and an adequate bladder cuff around the ureteral orifice. Additionally, we report the midterm oncological results for the treated group of patients. 

## 2. Materials and Methods

Between September 2007 and April 2011, 10 patients (7 men and 3 women) diagnosed with upper tract TCC were treated with this procedure. The median age was 68.5 years (range 48–81 years). Macroscopic hematuria was the most common clinical presentation. The patient characteristics are shown in Table 1. Patients with a previous history of bladder TCC were excluded. Additionally, no patient with a tumor of the juxtavesical or intramural ureter was treated using this approach. Transvesical laparoscopic circumferential detachment of the bladder cuff and en bloc mobilization of the last centimeters of the distal ureter were performed, followed by the closure of the bladder defect. This step was always the first part of the procedure. Subsequently, nephrectomy and removal of the specimen (en bloc kidney, ureter and bladder cuff) were performed either laparoscopically or using an open flank approach.

### 2.1. Operative Technique

 After the induction of general anesthesia, the patient was placed in a modified lithotomy position with the hips and knees flexed at approximately 50° and 30° degrees, respectively. A standard 21 Fr rigid cystoscope was inserted, and a thorough bladder inspection was performed to exclude any visible lesions. Both ureteral orifices were identified.

**Figure 1:**
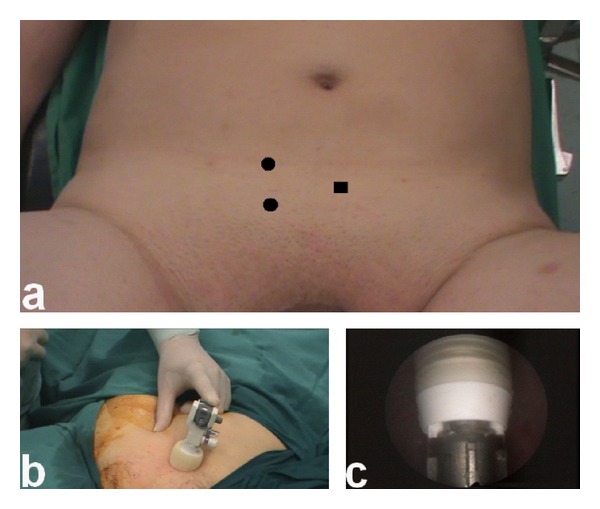
(a) Trocar arrangement for the excision of the left distal ureter. The mirror image of this configuration was used for the other side. (■) 10 mm camera port; (●) 5 mm working ports. (b) 10 mm camera port in place. (c) Cystoscopic view of the 10-mm port.

Three transvesical trocars arranged in a triangular configuration were used (Figure 1). After filling the bladder at its maximum capacity, the first trocar was placed in the bladder under cystoscopic visualization (Figure 1). The first trocar for the laparoscope was a 10 mm self-retaining balloon trocar (OMST10BT, Covidien, Norwalk, CT, USA). This trocar is a blunt tip trocar, and its insertion was facilitated by replacing its obturator with the obturator of a standard 10 mm blade trocar. The balloon of the trocar was inflated, and the sealing foam was pushed against the abdominal wall. This maneuver not only stabilized the bladder against the abdominal wall but also prevented leakage around the trocar. The 10 mm trocar was placed at the bladder dome lateral to the midline and toward the ureteral orifice to be excised (Figure 1). Two standard 5 mm dilating step trocars (VersaStep 5 mm, Covidien, Norwalk, CT, USA) were then inserted under cystoscopic guidance.

**Figure 2:**
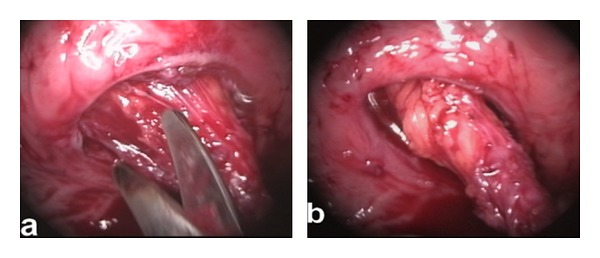
(a), (b) Sharp dissection and progressive mobilization of the distal ureter.

**Figure 3:**
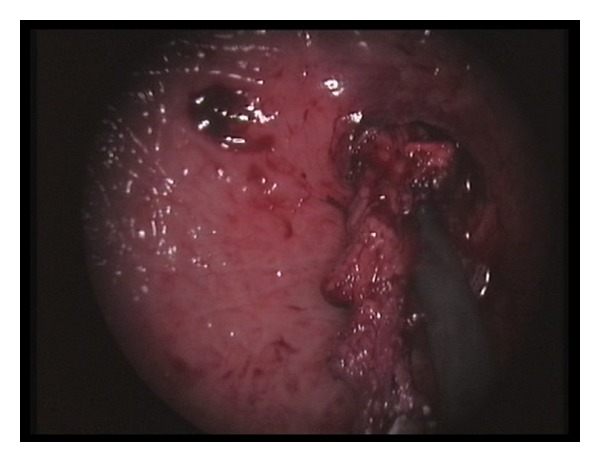
The ureter is completely dissected and freed from the bladder wall.

**Figure 4:**
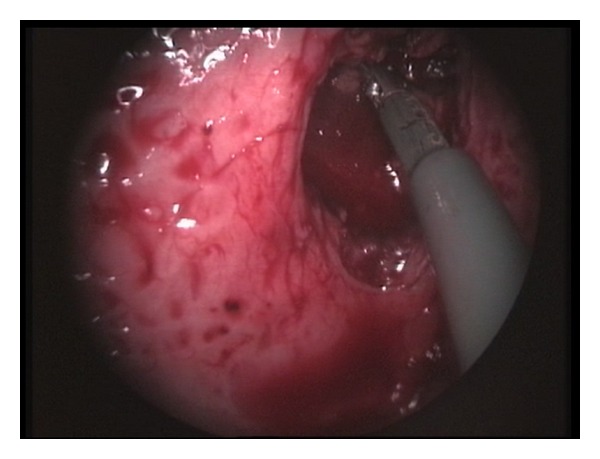
The mobilized distal ureter is placed outside the bladder. The bladder defect will be closed with running suture.

The bladder was emptied, and the cystoscope was removed. Pneumovesicum was established with CO_2_ insufflation at a pressure of 12 mmHg. Using a Maryland dissector on the surgeon's nondominant hand, the ureteral orifice was tented up, and a mucosal margin of approximately 1 cm around the orifice was circumferentially marked using diathermy. Using laparoscopic scissors, the orifice and the distal ureter were progressively dissected free from the bladder wall (Figure 2). To prevent urine leakage from the affected system, the distal ureter was sealed using either a sealing device (LigaSure 5 mm Covidien, Norwalk, CT, USA) or a 5 mm; clip applier. When the distal ureter was adequately mobilized and fully freed from the bladder wall, it was placed outside the bladder (Figures 3 and 4). Using a 2/0 Vicryl suture on a CV-23 needle, the bladder defect was closed in one layer using continuous suturing. Finally, the trocars were removed without closing the entry sites. A 16Fr Foley catheter was left in situ for one week.

The patient was then placed in a 90° lateral decubitus position, and either an open nephrectomy via a standard flank incision or a transperitoneal laparoscopic nephrectomy was performed. In both approaches, after mobilizing the ureter caudally, the previously detached juxtavesical ureter and bladder cuff were easily removed. In the open approach, the entire specimen was extracted through the flank incision. In the laparoscopic approach, the en bloc specimen was placed in a specimen retrieval bag. The incision at one of the port sites was extended appropriately, and the bag was removed.

## 3. Results

 The distal ureter and bladder cuff excision procedure was completed uneventfully in all cases. The operating time for distal ureter excision ranged from 55 to 120 minutes (median 82.5 minutes). This time was calculated from the insertion of the cystoscope to the removal of the transvesical trocars. The operating time decreased from case 1 through case 10 due to increased experience. Blood loss related to the excision of the distal ureter was minimal in all cases (<50 mL). Open nephrectomy via a standard flank approach was performed in the first two cases, whereas laparoscopic transperitoneal nephrectomy was performed in the last eight cases. No complications directly related to the pneumovesicum method were recorded. In one patient (case 2), a postoperative fever >38°C was recorded on the 2nd postoperative day. This fever resolved spontaneously. Cystography before catheter removal (on the 7th postoperative day) was performed in the first two cases without evidence of extravasation. This examination was not performed in the last eight patients, and no complications were recorded. The mucosal margins of the bladder cuff were negative in all ten cases.

The median follow-up duration for this series was 31 months (range 12–55 months). During the follow-up period, two patients died from the disease. Patient number 2 had a T3 renal pelvic tumor and presented with both nodal and distant metastases 6 months postoperatively. Patient number 8 had a T2 renal pelvic tumor and initially developed metastases at the paraaortic lymph nodes 12 months postoperatively. Both patients received chemotherapy but died 13 and 22 months after surgery, respectively. Noticeably none of the patients in this series developed local pelvic recurrences or pelvic lymph node metastases. A bladder tumor developed in three patients (30%) during the follow-up period. The tumors were found on the lateral bladder wall on the contralateral side with respect to the excised orifice in two patients and on the bladder dome in one patient. The intra- and postoperative data are summarized in Table 2.

## 4. Discussion

 Historical data have demonstrated the critical importance of proper distal ureteral excision due to the high incidence of recurrences in the ureteral stump and perimeatal bladder mucosa of patients treated with incomplete ureterectomy [7, 8]. In open surgery, transvesical, extravesical, and combined approaches have been described to accomplish complete distal ureterectomy with a bladder cuff. The transvesical approach requires a cystotomy, and the ureteral orifice with a 1 cm bladder cuff is completely mobilized from inside the bladder and removed with the entire nephroureterectomy specimen. Although the bladder is opened, this approach is the most reliable. In the extravesical approach, a formal cystotomy is not required. Instead, the ureter is tented up, and a portion of the bladder wall along with the distal ureter is removed after placing a clamp. The less cumbersome extravesical approach does not ensure the complete removal of the intramural portion of the ureter and theoretically carries a risk of contralateral injury from excessive traction. Strong and Pearse [8] reported nine cases in which the open extravesical approach was used. On subsequent cystoscopy and retrograde ureterography, all nine patients were noted to have a ureteral orifice and an intramural ureter. Two of the nine patients had tumor recurrence in the ureteral stump.

In the era of laparoscopic surgery, there have been attempts to duplicate both open techniques with various modifications. The laparoscopic extravesical approach was among the first attempted despite the drawbacks of the open extravesical technique described before. Obviously, this technique was performed because, as in open surgery, the laparoscopic extravesical approach is technically less demanding. Shalhav et al. [9] have described a laparoscopic approach combined with a modified transurethral resection of the orifice. In their technique, a ureteral catheter with an occlusion balloon is first placed, to prevent tumor seeding prior to the laparoscopic nephroureterectomy. Subsequently, the bladder cuff is created transurethrally until 1 cm of the ureteral tunnel is developed. Then, the distal ureter is dissected laparoscopically, and the bladder cuff is divided using a laparoscopic endoscopic gastrointestinal anastomosis (Endo GIA) stapler. In the past, Hattori et al. [10] used a completely laparoscopic extravesical stapling technique. The distal ureter, and bladder cuff were transected with a stapler after dissecting the bladder muscle along the ureter down to its intramural portion. Although this technique is simple and reduces the operative time, stone formation was found to occur later, and in some cases, the orifice was not actually excised. Therefore, this group has modified their technique and now they dissect the ureter down to the bladder and open the bladder after placing a stay suture. Under laparoscopic visualization, the bladder mucosa is incised around the orifice, and the distal ureter is removed with a bladder cuff. The opened bladder wall is then closed with running stitches [11]. In general, laparoscopic extravesical stapling of the distal ureter and bladder cuff is an attractive approach because the urinary tract is not opened, and tumor spillage is minimized. Additionally, this technique is rapid, especially in the hands of experienced laparoscopists. The disadvantages include the potential for positive surgical margins, the failure to remove the ipsilateral orifice, and a small but nonnegligible risk of compromising the contralateral orifice. Matin and Gill [12] evaluated the patterns of recurrence and survival for the various forms of bladder cuff control in a retrospective study. They demonstrated that positive margins were more frequently associated with a laparoscopic stapling approach than with either the transvesical or open techniques. Most importantly, the laparoscopic stapling approach was also associated with poorer recurrence-free survival. Tsivian et al. [13] have described a purely laparoscopic nephroureterectomy technique that utilizes two additional trocars in the ipsilateral lower abdomen after a standard transperitoneal nephrectomy. Caudal ureteral dissection continues until the detrusor muscle fibers at the ureterovesical junction are identified. The ureter is then retracted upward, tenting up the bladder wall. The bladder cuff is excised using a 10 mm LigaSure Atlas device, which seals the bladder defect. This method does avoid some of the disadvantages associated with the extravesical stapling technique. However, at least theoretically, this method does not address the issue of possible incomplete distal ureteral resection.

Another group of techniques based on the transvesical approach was also introduced in an effort to mimic the reliable open transvesical excision technique. Gill et al. [14] have described a novel laparoscopic technique that involves the use of two 2 mm transvesical suprapubic trocars and a ureteral stent in the ipsilateral ureter. The ureter is tented upward; a loop ligature is placed around the stent, creating a closed system, and a Collin's knife is then used to excise the ureteral orifice. A technique resembling this technique has been reported by Ahlawat and Gautam [15], in which only one transvesical suprapubic 5 mm port is used. A transurethral resectoscope is used to make a full-thickness incision in the bladder cuff around the ureteric orifice from 1 o'clock to 11 o'clock. A grasper inserted through the transvesical suprapubic port is then used to retract the ureter to complete the incision in the bladder cuff overlying the anterior aspect of the ureteric orifice. The ureter is subsequently sealed with a clip applied through the port. Recently, a very similar technique was described for a series of six patients by Zou et al. [16]. Instead of a 5 mm port, they utilized a 10 mm port placed transvesically after pneumovesicum had been established [16]. The excision was performed with a Collin's knife, and a hem-o-lok clip was applied through the 10 mm transvesical port to seal the system. Pathak et al. [17] reported a modification of the “pluck” technique in which a Collin's knife is used to incise the bladder deep into the muscle with a margin of <5 mm. A 5 mm laparoscopic hem-o-lok clip is inserted via the straight working channel of the cytoscope into the bladder and applied across the intramural ureter. Following patient repositioning, either a retroperitoneal or transperitoneal laparoscopic nephroureterectomy is performed. In hand-assisted LNU, various modifications of the “pluck” technique have been used [18–20]. In general, the surgeon's intra-abdominal hand facilitates bladder cuff and ureteral excision, which is performed using a Collin's knife inserted transurethrally [18] or through a nephroscope placed in the bladder suprapubically [19], or using a flexible cystoscope combined with a 5F electrode on cutting current [20]. When a nephroscope is used, it is inserted through a standard 10 mm laparoscopic trocar placed extraperitoneally directly into the bladder [19]. The primary disadvantage of all of the previously described transvesical techniques is that neither the ureteral defects nor the defects created by the transvesical ports were closed, but postoperative urine extravasation was limited [14–20]. In 2007, Cheng et al. [6] reported one case in which a pure transvesical laparoscopic excision was performed. Three pediports were placed in the bladder, pneumovesicum was established, and after excision of the orifice with a bladder cuff, the ureteral defect was closed with freehand suturing. This technique was the first to completely duplicate the traditional open transvesical approach. However, the trocar sites were not closed. A bladder catheter was left in situ for 7 days.

We adopted this technique almost immediately after its publication, with some minor modifications. First, a 10 mm self-retaining balloon trocar is used, which accommodates the standard 10 mm laparoscope. The primary reason for this change was the unavailability of a 5 mm laparoscope in our department when this technique was first applied. However, we have found that the balloon trocar, despite its larger diameter, stabilizes the bladder dome against the abdominal wall and minimizes leakage around the entry site. Second, instead of the Pediports, 5 mm step trocars are used, which are more versatile and more stable, preventing inadvertent exit from the bladder. Guzzo et al. [21] have also reported a modification of the technique by Cheng et al. [6]. Guzzo et al. used a modified lateral decubitus position with the hips supine. Laparoscopic nephroureterectomy was performed first, followed by excision of the distal ureter without need for patient repositioning, as the patient's hips are already flat on the operating table. Additionally, the three trocars utilized for the procedure were equally spaced and were placed into the bladder transversely two finger widths above the pubic bone [21]. The advantages of the pneumovesicum technique are reliable excision of the distal ureter and bladder cuff, constant visualization and, therefore, protection of the contralateral orifice, and closure of the bladder defect, which minimizes urine spillage. The disadvantages include the fact that the trocar sites are not closed, the patient requires repositioning for the nephrectomy—unless the technique described by Guzzo et al. [21] is used—and the operating time is most likely longer than those of other transvesical techniques. Although the trocar sites are not closed, this lack of closure does not represent a substantial problem because the entry points are located at the bladder dome and, if adequate urine drainage is maintained, urine extravasation is practically absent [6]. We have not had problems with urine extravasation despite the fact that we have used a 10 mm trocar for the camera. Most likely, the next step is the technique reported by Sotelo et al. [22], in which a single-port device was inserted transvesically and pneumovesicum established. Distal ureterectomy was performed, and the bladder defect was closed using intracorporeal suturing with extracorporeal knots. As described in their case report, these authors performed a LESS nephroureterectomy first and distal ureteral excision second. The advantage of this technique over the standard pneumovesicum method is the fact that the anterior cystotomy for the placement of the single-port device was also closed. 

From an oncological point of view, the pneumovesicum method does not seem to adversely influence the final outcome. Both deaths in our series were most likely related to the advanced stage of the disease rather than the method employed for distal ureter excision. Similarly, bladder recurrences were more or less within the expected rate for bladder recurrence in upper tract urothelial carcinoma [5, 12, 13]. The group that first reported the pneumovesicum method [6] has also recently reported the midterm oncological results of their series [23]. During a median follow-up period of 46 months, they had one (10%) systemic recurrence and a 40% bladder recurrence rate. Our results are generally in accordance with theirs, demonstrating that the technique of laparoscopic transvesical resection of the en bloc bladder cuff and distal ureter is reliable and ontologically safe, at least in the midterm. 

## Figures and Tables

**Table 1:** Patients' characteristics.

Patient	Sex	Age	Tumor location (side)	Clinical presentation
1	M	64	Renal pelvis (Lt)	Hematuria
2	M	73	Renal pelvis (Lt)	Hematuria
3	F	68	Upper ureter (Rt)	Hematuria, renal colic
4	M	79	Upper calyx (Lt)	Hematuria
5	M	55	Renal pelvis and middle calyx (Lt)	Hematuria
6	M	69	Middle ureter (Lt)	Hematuria, hydronephrosis
7	M	48	Lower calyx (Rt)	Hematuria
8	F	64	Renal pelvis (Lt)	Hematuria
9	F	81	Renal pelvis (Rt)	Hematuria
10	M	77	Renal pelvis (Rt)	Incidental finding

M: male, F: female, Rt: right, Lt: left.

**Table 2:** Intra- and postoperative data.

Patient	Operating time* (minutes)	Pathological stage/grade	Followup (months)	Bladder recurrence	Outcome
1	120	Ta/grade 1	55	No	Alive
2	113	T3/grade 3	13	No	Dead
3	104	T1/grade 2	48	Yes	Alive
4	97	Ta/grade 1	41	No	Alive
5	85	T1/grade 2	36	Yes	Alive
6	80	T2/grade 2	33	No	Alive
7	73	Ta/grade 1	29	No	Alive
8	75	T2/grade 2	22	No	Dead
9	68	T1/grade 2	19	Yes	Alive
10	55	Ta/grade 2	12	No	Alive

*Refers to the transvesical procedure.

## References

[1] Clayman RV, Kavoussi LR, Figenshau RS, Chandhoke PS, Albala DM (1991). Laparoscopic nephroureterectomy: initial clinical case report. *Journal of Laparoendoscopic Surgery*.

[2] McDougall EM, Clayman RV, Elashry O (1995). Laparoscopic nephroureterectomy for upper tract transitional cell cancer: the Washington University experience. *Journal of Urology*.

[3] Stifelman MD, Hyman MJ, Shichman S, Sosa RE (2001). Hand-assisted laparoscopic nephroureterectomy versus open nephroureterectomy for the treatment of transitional-cell carcinoma of the upper urinary tract. *Journal of Endourology*.

[4] Waldert M, Remzi M, Klingler HC, Mueller L, Marberger M (2009). The oncological results of laparoscopic nephroureterectomy for upper urinary tract transitional cell cancer are equal to those of open nephroureterectomy. *BJU International*.

[5] Stewart GD, Humphries KJ, Cutress ML (2011). Long-term comparative outcomes of open versus laparoscopic nephroureterectomy for upper urinary tract urothelial-cell carcinoma after a median follow-up of 13 years. *Journal of Endourology*.

[6] Cheng CW, Ng CF, Mak SK (2007). Pneumovesicum method in en-Bloc laparoscopic nephroureterectomy with bladder cuff resection for upper-tract urothelial cancer. *Journal of Endourology*.

[7] Strong DW, Pearse HD, Tank ES, Hodges CV (1976). The ureteral stump after nephroureterectomy. *Journal of Urology*.

[8] Strong DW, Pearse HD (1976). Recurrent urothelial tumors following surgery for transitional cell carcinoma of the upper urinary tract. *Cancer*.

[9] Shalhav AL, Elbahnasy AM, Mcdougall EM, Clayman RV (1998). Laparoscopic nephroureterectomy for upper tract transitional-cell cancer: technical aspects. *Journal of Endourology*.

[10] Hattori R, Yoshino Y, Gotoh M, Katoh M, Kamihira O, Ono Y (2006). Laparoscopic nephroureterectomy for transitional cell carcinoma of renal pelvis and ureter: nagoya experience. *Urology*.

[11] Hattori R, Yoshino Y, Komatsu T, Matsukawa Y, Ono Y, Gotoh M (2009). Pure laparoscopic complete excision of distal ureter with a bladder cuff for upper tract urothelial carcinoma. *World Journal of Urology*.

[12] Matin SF, Gill IS (2005). Recurrence and survival following laparoscopic radical nephroureterectomy with various forms of bladder cuff control. *Journal of Urology*.

[13] Tsivian A, Benjamin S, Sidi AA (2007). A sealed laparoscopic nephroureterectomy: a new technique. *European Urology*.

[14] Gill IS, Soble JJ, Miller SD, Sung GT (1999). A novel technique for management of the en bloc bladder cuff and distal ureter during laparoscopic nephroureterectomy. *Journal of Urology*.

[15] Ahlawat RK, Gautam G (2011). Suprapubic transvesical single-port technique for control of lower end of ureter during laparoscopic nephroureterectomy for upper tract transitional cell carcinoma. *Indian Journal of Urology*.

[16] Zou X, Zhang G, Wang X (2011). A one-port pneumovesicum method in en Bloc laparoscopic nephroureterectomy with bladder cuff resection is feasible and safe for upper tract transitional cell carcinoma. *BJU International*.

[17] Pathak S, Watcyn-Jones T, Doyle D, Oakley N (2008). Laparoscopic nephro-ureterectomy for upper tract urothelial cancer: cystoscopic closed system pluck. *Annals of the Royal College of Surgeons of England*.

[18] Wong C, Leveillee RJ (2002). Hand-assisted laparoscopic nephroureterectomy with cystoscopic en bloc excision of the distal ureter and bladder cuff. *Journal of Endourology*.

[19] Gonzalez CM, Batler RA, Schoor RA, Hairston JC, Nadler RB (2001). A novel endoscopic approach towards resection of the distal ureter with surrounding bladder cuff during hand assisted laparoscopic nephroureterectomy. *Journal of Urology*.

[20] Vardi IY, Stern JA, Gonzalez CM, Kimm SY, Nadler RB (2006). Novel technique for management of distal ureter and en block resection of bladder cuff during hand-assisted laparoscopic nephroureterectomy. *Urology*.

[21] Guzzo TJ, Schaeffer EM, Allaf ME (2008). Laparoscopic radical nephroureterectomy with en-bloc distal ureteral and bladder cuff excision using a single position pneumovesicum method. *Urology*.

[22] Sotelo R, Ramírez D, Carmona O (2011). A novel technique for distal ureterectomy and bladder cuff excision. *Actas Urologicas Espanolas*.

[23] Mak SK, Ng CF, Chan ESY, Yip SKH, Cheng CW, Wong WS (2011). Pneumovesicum approach to en-Bloc laparoscopic nephroureterectomy with bladder cuff excision for upper tract urothelial cancer: midterm oncological results. *Journal of Endourology*.

